# Peripheral Nervous System Neuropathology and Progressive Sensory Impairments in a Mouse Model of Mucopolysaccharidosis IIIB

**DOI:** 10.1371/journal.pone.0045992

**Published:** 2012-09-25

**Authors:** Haiyan Fu, Julianne D. Bartz, Robert L. Stephens, Douglas M. McCarty

**Affiliations:** 1 Center for Gene Therapy, The Research Institute at Nationwide Children’s Hospital, Columbus, Ohio, United States of America; 2 Department of Pediatrics, The Ohio State University, Columbus, Ohio, United States of America; 3 Department of Physiology and Cell Biology, The Ohio State University, Columbus, Ohio, United States of America; University of Edinburgh, United Kingdom

## Abstract

The lysosomal storage pathology in Mucopolysaccharidosis (MPS) IIIB manifests in cells of virtually all organs. However, it is the profound role of the neurological pathology that leads to morbidity and mortality in this disease, and has been the major challenge to developing therapies. To date, MPS IIIB neuropathologic and therapeutic studies have focused predominantly on changes in the central nervous system (CNS), especially in the brain, and little is known about the disease pathology in the peripheral nervous system (PNS). This study demonstrates characteristic lysosomal storage pathology in dorsal root ganglia affecting neurons, satellite cells (glia) and Schwann cells. Lysosomal storage lesions were also observed in the myoenteric plexus and submucosal plexus, involving enteric neurons with enteric glial activation. Further, MPS IIIB mice developed progressive impairments in sensory functions, with significantly reduced response to pain stimulation that became detectable at 4–5 months of age as the disease progressed. These data demonstrate that MPS IIIB neuropathology manifests not only in the entire CNS but also the PNS, likely affecting both afferent and efferent neural signal transduction. This study also suggests that therapeutic development for MPS IIIB may benefit from targeting the entire nervous system.

## Introduction

Mucopolysaccharidosis (MPS) IIIB is a lysosomal storage disease caused by the autosomal recessive defect of *α*-*N*-acetylglucosaminidase (NaGlu) gene [1]. NAGLU is a lysosomal enzyme that is essential in the stepwise metabolic degradation of heparan sulfate (HS), a class of biologically important glycosaminoglycans (GAGs). Mutations are highly heterogenic among patients with MPS IIIB [1], [2], [3]. The lack of NaGlu activity results in the accumulation of HS and HS-derived oligosaccharides in lysosomes of cells in virtually all organs, especially cells throughout the central nervous system (CNS), including neuronal and non-neuronal cell-types, although not all CNS cells are affected [4]. Patients with MPS IIIB appear normal at birth, but develop a progressive and severe neurological disorder, though somatic manifestations of MPS IIIB are relatively mild compared to other types of MPS. High mortality and premature death are typical in MPS IIIB patients. Only Palliative treatment is available for the disease.

It is known that the lysosomal storage pathology of MPS IIIB is global throughout the CNS and neuropathology studies have focused predominantly on changes in the brain, though previous studies showed lysosomal storage lesions in all parts of the CNS in mice and dogs [4], [5]. Further, it is commonly accepted that in the brain, multiple factors contribute to the neuropathology, secondary to the primary pathology- the lysosomal storage of HS-GAGs [4], [6], [7], [8], [9], [10], [11], [12], [13], [14]. The complex MPS IIIB neuropathology has been shown to involve impaired metabolism, inflammation, neurodegeneration, reactive oxygen species and tauopathy. A correlation between high efficiency of GAG synthesis and a severe phenotype was recently reported in patients with MPS, including MPS II, IIIA and IIIB [15]. However, little is known regarding the lower CNS and PNS neuropathology. A recent study revealed thinning of the outer nuclear layer and inclusions in pigmented epithelium of the retina, and loss of hair cells in the inner ear and histologic abnormalities in the middle ear, and showed impaired vision and hearing in MPS IIIB mice [16]. In MPS IIIB gene therapy studies, an intracisternal and/or a systemic delivery of adeno-associated viral vector following mannitol pretreatment to disrupt the blood-brain-barrier yielded improvements in longevity and cognitive function [17], [18], [19], [20]. However, significantly improved motor function in the rotarod assay was observed only when the systemic vector delivery was also involved, suggesting that deficiencies in motor function can only be effectively corrected with gene delivery to both the CNS and the periphery. This may be supported by a recent study showing that combining intracranial AAV vector delivery and syngeneic bone marrow transplant resulted in improved therapeutic impact in MPS IIIB mice, compared to each approach only [21].

The present study demonstrates that the MPS IIIB neuropathology is present throughout the entire nervous system, including the CNS and PNS. We demonstrate characteristic lysosomal storage in the dorsal root ganglion (DRG) and enteric nervous system (ENS) in an MPS IIIB mouse model. Our data also showed significant impairment in pain sensory capacity as the disease progresses. These data suggest the potential role of the PNS neuropathy in MPS IIIB.

## Materials and Methods

### MPS IIIB Mice

The mouse model of MPS IIIB, generated by homologous recombination [4], was maintained on an inbred background (C57BL/6) of backcrosses of heterozygotes. The MPS IIIB mice resemble the human disease, have no detectable NaGlu activity in any tissue, and exhibit characteristic lysosomal storage pathology and clinical disorders. The mice were housed in the Vivarium at the Research Institute at Nationwide Children’s (NCRI). All care and procedures were in accordance with the *Guide for the Care and Use of Laboratory Animals* [DHHS Publication No. (NIH) 85-23. The genotypes of progeny mice were identified by PCR, using primers targeting undisrupted mouse NaGlu Exon 6: 5′-TGGACCTGTTTGCTGAAAGC (sense) and 5′-CAGGCCATCAAATCTGGTAC (anti-sense), and the transgene (neomycin): 5′-TGGGATCGGCCATTGAACAA (sense) and 5′-CCTTGAGCCTGGCGAACAGT (anti-sense). MPS IIIB mice and their wild type (wt) littermates were used in this study. The animal studies have been approved by the IACUC committee in the Research Institute at Nationwide Children’s Hospital (IACUC#: 04804AR).

### Histopathology and Transmission Electron Microscopy (TEM)

For tissue sample collection, age-matched MPS IIIB and wt mice (n = 4 each) were anesthetized with an intraperitoneal injection of avertin (2.5%, 0.3–0.4 mg/g body weight), and were then perfused transcardially with cold PBS (0.1 M, pH 7.4) followed by 4% paraformaldehyde in phosphate buffer (0.1 M, pH 7.4). Spinal cord and small intestine samples were fixed in 4% paraformaldehyde and processed for paraffin sections (4 µm). The sections were then processed for histology staining using toluidine blue (1%), which does not stain GAGs.

Spinal cord, spinal nerve and samples of cervical, thoracic and lumbar DRG (n = 4 each) were fixed in 2.5% glutaraldehyde overnight and processed for plastic sections (1–2 µm). The samples were collected and processed at the same time. The sections were then assayed for TEM and/or histopathology with toluidine blue staining.

### Immunofluorescence (IF)

Indirect IF was performed on the spinal cord and DRG paraffin sections (4 µm)(n = 4/group) following the protocol provided by the manufacturers, using a primary antibody against glial fibrillary acidic protein (GFAP)(Millipore), Iba 1 (Abcam), or LAMP-1(Novus Biologicals), and secondary antibodies conjugated with AlexaFluo^488^ or AlexaFluo^568^ (Invitrogen). The sections were visualized under a fluorescent microscope. GFAP- or Iba1-positive cells in grey matter of spinal cord in an area of 300×430 µm were counted on cervical, thoracic and lumbar sections from 6 mice per group. Cell counting was performed on 3 sections/level/mouse.

### qRT-PCR

Total RNA samples from lumbar spinal cord were assayed for CD45 expression by Real-time qRT-PCR, using a pair of primers for murine CD45 gene, as previously described. [12] A pair of primers for murine GAPDH (glyceraldehyde-3-phosphate dehydrogenase) gene was used as internal control. [12] Superscript First-strand Synthesis System for RT-PCR (Invitrogen) was used for cDNA synthesis. The qRT-PCR was performed by SYBR Green PCR Master Mix (Applied Biosystems). Comparative threshold (CT) method was used for data analysis. Data were expressed as relative quantitation of gene expression (2-ddCt), in the spinal cord from MPS IIIB vs. wt mice.

### Touch Test

Touching for changes in the response to tactile stimulation was performed using touch test sensory monofilaments (Semes-Weinstein von Frey Filament, North Coast Medical) [22]. Mice (n = 10/group, male:female = 1∶1) were acclimated over 1 h to an inverted elevated transparent cage with 0.25-inch wire mesh bottom. Both rear paws were tested. The filament was placed on the middle of the foot pad for 6 sec, with enough pressure to just bend the filament. Each rear paw was tested 5 times with each filament, starting with filament 3.61, followed by 4.31, 4.56 and 5.07 g (gram: touch force). The response measures were taken as positive if the mouse withdrew its foot.

### Tail Flick Test

Algesic response to thermal stimuli in mice was measured with the tail flick test [23] using an automated tail-flick analgesia meter (Columbus Instruments) following the procedures recommended by the manufacturer. The assay was performed when mice were 2 mo (n = 10, m:f = 1∶1), 3 mo (n = 10, m:f = 1∶1), 4 mo (n = 15, m:f = 8∶7), and 6 mo old (n = 10, m:f = 1∶1 ). Age-matched wt littermates were used as controls (n = 10/group, m:f = 1∶1). Mice were placed in a clear restraining tube and the tail was placed freely in the grove of the tail holder. Immediately after a 90-second habituation, radiant heat from a beam of building light source was focused on the ventral surface of the tail and the time for the mouse to flick its tail away from the surface was automatically recorded. A 10-seconds cut-off time was employed to prevent tissue damage. Each mouse was given 2 trials, separated by 1 h interval, data were average of the results from 2 trials.

### Hot-plate Test

The thermal nociceptive threshold in mice was assessed using a hot-plate analgesia apparatus (Columbus Instruments) [24], following the procedures recommended by the manufacturer. The assay was performed when mice were 2 mo (n = 10, m:f = 1∶1), 3 mo (n = 10, m:f = 1∶1), 4 mo (n = 15, m:f = 8∶7), and 6 mo old (n = 10, m:f = 1∶1). Age-matched wt littermates were used as controls (n = 10/group, m:f = 1∶1). Mice were placed on a hot plate thermostatically set at 55.0°C. The latency of first licking or kicking of the fore or hind paw was recorded. A cut-off time of 60 seconds was employed to avoid tissue damage. Each mouse was given 2 trials, separated by 1 h interval, data were average of the results from 2 trials.

### Statistics

Means, standard deviation and student *t*-test were used to analyze quantitative data. Unquantified data from touch tests were analyzed by chi-square test and logistic regression with repeated measurements (GENMOD) using SAS9.2.

## Results

### Profound Lysosomal Storage Manifestation in DRG in MPS IIIB Mice

**Figure 1:**
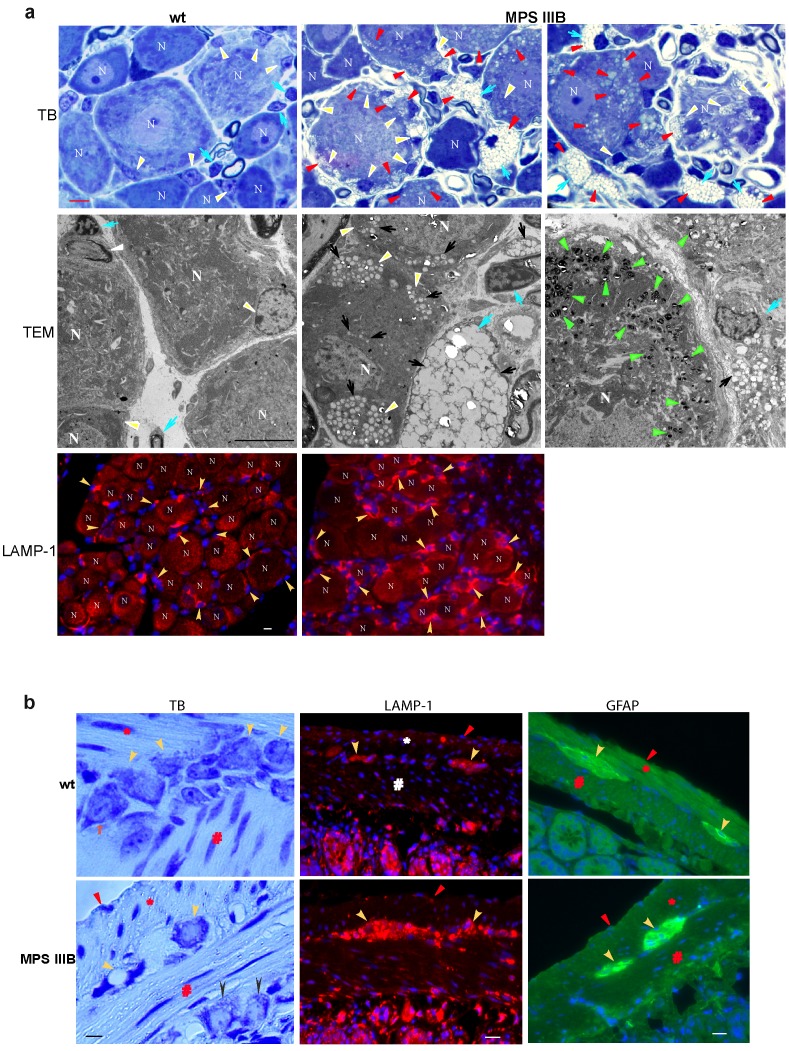
Neuropathology in the dorsal root ganglion and enteric nervous system MPS IIIB mice. DRG (a) and small intestine (b) isolated from 6 mo old MPS IIIB mice were processed for histopathology using toluidine blue (TB) staining, TEM and IF for LAMP-1 or GFAP. a. DRG: Top panel: TB staining,; middle panel: TEM; bottom panel: IF for LAMP-1. N: neurons; white arrowheads: satellite cells; blue arrows: Schwann cells; red arrowheads: lysosomal storage lesions; black arrows: low density material storage under TEM; green arrowheads: zebra bodies. Red fluorescence: LAMP-1-positive signal (yellow arrowheads). b. Intestine: Left panel: TB staining. Middle panel: IF for LAMP-1 (red fluorescence). Right panel: IF for GFAP (green fluorescence). *: longitudinal muscle layer; #: circular muscle layer; yello arrowheads: neurons of myenteric plexus; black arrowheads: neurons of submucosal plexus; red arrowhead: peritoneal surface. Scale bars: 20 µm.

Histopathology and TEM on DRG sections were performed to determine whether the lysosomal storage pathology affects the PNS in MPS IIIB mice in comparison with wt mice (n = 6/group, 6 mo old). Histopathology staining with toluidine blue showed wide spread lysosomal storage lesions in virtually all neurons, satellite cells (PNS glia) and Schwann cells in DRG from MPS IIIB mice (**Fig. 1a**). Under TEM, the storage lesions in the DRG exhibited as low density vesicles and zebra bodies (**Fig. 1a** **)**, which are known characteristic storage pathology in the CNS of animals and patients with MPS IIIB. Our data also showed increase in LAMP-1 staining in DRGs (**Fig. 1a**). There was no observable pathological difference in tested cervical, thoracic and lumbar DRGs. Using TEM, in these mice, we also observed a significant decrease in endoneurial collagen fibril structures surrounding myelinated neuritis in the DRGs (**data not shown**), indicating a degenerative pathology in the DRG. TEM also showed the destruction of myelin sheath in the spinal nerves from MPS IIIB mice (Supplemental data), suggesting possible demyelination in the PNS. In addition, the myelin sheath destruction is ubiquitous in the spinal nerves, suggesting the involvement of both DRG sensory neurons and spinal cord motor neurons. Our data demonstrate a PNS component of neuropathology.

### Lysosomal Storage and Glial Activation in Enteric Nervous System (ENS) in MPS IIIB Mice

Histopathology using toluidine blue staining was also used to probe lysosomal storage lesions in the intestine. We observed vacuoles, characteristic lysosomal storage lesions, in the neurons in myenteric plexus and submucosal plexus (**Fig. 1b**) and enteric glial cells (data not shown) throughout the intestine in MPS IIIB mice. Increase in LAMP-1 signals in enteric plexus was also observed, supporting the lysosomal storage manifestation in the enteric nervous system. Further, IF staining showed an increase in GFAP signals in all the myenteric plexus and submucosal plexus in MPS IIIB mice that were observed (**Fig. 1b**), suggesting enteric glial activation secondary to the lysosomal storage pathology. These data further support a widespread PNS involvement in MPS IIIB neuropathology.

### Progressive Functional Sensory Impairment in MPS IIIB Mice

To assess whether MPS IIIB affects sensory function, mice were tested for response to nociceptive stimuli by microfilament touch test (n = 10/group,), tail flick and hot plate assays (n = 10–15/group).

**Figure 2:**
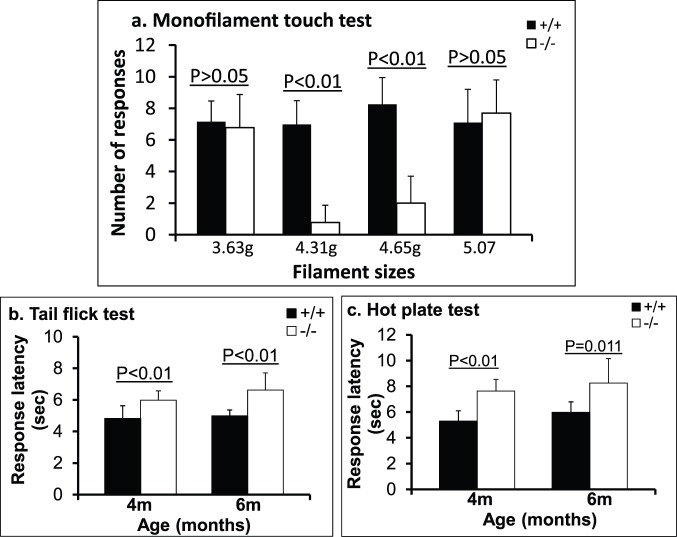
Loss of tactile and thermal nociceptive response in MPS IIIB mice. MPS IIIB (−/−) and wt (+/+) littermates were tested for sensory functions. a. Monofilament touch test (n = 10/group, 6 mo of age): the unquantified data were analyzed using chi-square test and logistic regression with repeated measurements. b. Tail-flick assay. c. Hot plate test. 4 mo old MPS IIIB mice: n = 15/group; wt and 6 mo-old MPS IIIB mice: n = 10/group. Data are means±sd.

Tactile testing showed that 6 mo old MPS IIIB mice were significantly less responsive to hind paw stimulation with 4.13 g (P<0.001) and 4.65 g (P<0.001) microfilaments on their feet (**Fig. 2a**), compared to age-matched wt mice (n = 10/group, see Methods). There were no significant differences in the responsiveness to hind paw tactile stimulation with 3.61 g and 5.07 g filaments between MPS IIIB and wt mice. These results suggest that the observed differences with 4.13 g and 4.65 g may be due to specific sensory defects but not due to the effects of motor deficiencies on the outcome.

Tail flick and hot plate tests were performed in mice at 2, 3, 4 and 6 mo of age (n = 10–15/group, see Methods). MPS IIIB mice exhibited significant increase in the latency to respond to heat stimuli in the tail flick (**Fig. 2b**) and hot plate assays (**Fig. 2c**), when they were 4 mo of age or older. The increase in tail flick and hot plate latency in these mice became detectable when they were 4 mo old and appeared to increase further at 6 mo of age, compared with wt mice. We did not observe detectable changes in tail flick and hot plate latencies in the MPS IIIB mice when they were 2–3 mo old (data not shown). These data indicate that MPS IIIB neuropathology impairs the sensory function in these animals, leading to the progressive loss of sensation to tactile and thermal nociceptive stimuli.

### Global and Differential Lysosomal Storage Pathology, Astrocytosis, Microglia Activation and Demyelination in the Spinal Cord in MPS IIIB Mice

To assess neuropathological manifestations in lower CNS, the spinal cord samples from 6 mo old MPS IIIB and wt mice (n = 6/group) were assayed by histopathology, IHC and transmission electron microscopy.

**Figure 3:**
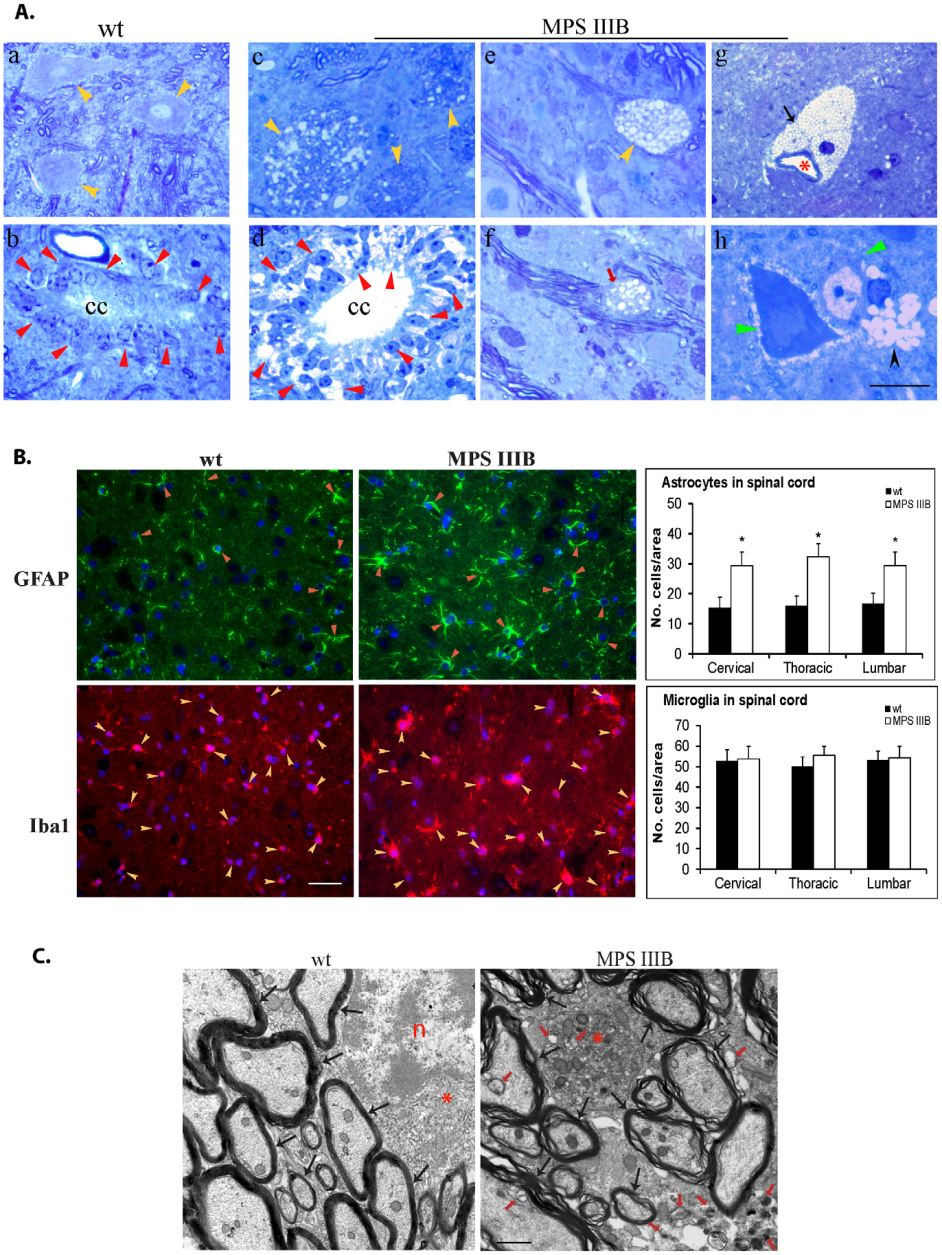
Lysosomal storage pathology, astrocytosis and demyelination in the spinal cord of MPS IIIB mice. Spinal cord samples from 6 mo old MPS IIIB mice were processed for paraffin sections (4 µm) and assayed by toluidine blue staining for histopathology (A), immunohistochemistry for GFAP and Iba1 (B) and transmission electron microscopy (C). A (lumbar). a-b: wt; c-h: MPS IIIB; yellow arrowheads: motor neurons (a, c, e); red arrowheads: Ependymal calls; cc: central canal (b, d); red arrow: oligodendrocyte (f); black arrow: pericyte; *: capillary (g); green arrowhead: sensory neurons (h); black arrowhead: microglia; scale bar = 20 µm. B. IF images: red arrowheads: GFAP-positive astrocytes and processes (green fluorescence). yellow arrowheads: Iba1-positive cells (red fluorescence). Scale bar = 50 µm. GFAP- or Iba1-positive cells were counted in an area of 300×430 µm on cervical, thoracic and lumbar sections from 6 mice per group. Cell counting was performed on 3 sections/level/mouse. No. cells: means±sd. C. TEM images (lumbar): black arrows: myelinated exons and synapses; *: cytoplasm of oligodendrocytes; n: nucleus of oligodendrocyte; red arrows: lysosomal storage lesions. Scale bar = 500 nm.

Histology staining with toluidine blue showed global and differential lysosomal storage pathology throughout the spinal cord in MPS IIIB mice (**Fig. 3A**). The lysosomal storage lesions were observed in a broad range of cells in the spinal cord, including neurons (**Fig. 3A** c & e), ependymal cells (**Fig. 3A** d), oligodendrocytes (**Fig. 3A** f), pericytes (**Fig. 3A** g), and microglia (**Fig. 3A** h). Lysosomal storage lesions appeared in all motor neurons in the ventral horn (**Fig. 3A** **c, e**) and in lateral horn neurons (data not shown), but not sensory neurons in the dorsal horn, even though adjacent microglia exhibited profound lysosomal accumulation pathology (**Fig. 3A** h). The characteristics of lysosomal storage pathology were consistent at different levels throughout the spinal cord. These results suggest the potential for damage of lower motor and autonomic functions and the potential role of spinal cord pathology in the neurological disease of MPS IIIB.

The IF staining showed significant increases in the intensity of GFAP signals and the number of GFAP-positive cells in both grey matter and white matter throughout the spinal cord in MPS IIIB mice compared to wt (**Fig. 3B**). Importantly, the GFAP-positive cells in the grey matter were protoplasmic in MPS IIIB mice and fibrous in wt animals (**Fig. 3B**). These data suggest the activation of astrocytes in the spinal cord of MPS IIIB mice. Using IF for IbaI, we observed increases in the intensity and the sizes of IbaI-positive cells throughout the spinal cord of MPS IIIB mice compared to wt animals (**Fig. 3B**), indicating widespread microglia activation, although there appeared no difference in the number of Iba1-positive cells in the spinal cord in MPS IIIB and wt mice (**Fig. 3B**). Increase in Iba1 staining was also seen in the spinal cord from 1 mo old MPS IIIB mice, indicating early emergence of microglial activation. In addition, qRT-PCR showed significant increase in the expression of CD45 in the spinal cord in 1 mo (1.7 fold) and 6 mo (2.1 fold) old MPS IIIB mice (data not shown), compared to wt, further supporting microglia activation. These results indicate a heightened inflammatory status in MPS IIIB mouse spinal cord.

Using TEM, we observed the destruction of myelin sheath throughout the spinal cord of MPS IIIB mice (**Fig. 3C**), suggesting that demyelination not only occurs in the brain, but also in the lower CNS in this disease.

## Discussion

To date, research on MPS IIIB neuropathology and therapeutic development have been predominantly focused on the CNS, especially the brain, and little is known regarding the pathological changes in other aspects of the nervous system. We demonstrate here that the neuropathology is manifested not only in the CNS, but also in the dorsal root ganglion and enteric nervous system, supporting the notion that the disease may affect a majority of, if not all, components of the nervous system. Because of the essential role of the DRG neurons in the afferent sensory signal (touch, stretch, temperature and pain) transduction, it is not surprising that the profound lysosomal storage pathology in MPS IIIB leads to the functional sensory impairments. This is supported by a recent study showing impaired vision and hearing in MPS IIIB mice [16], [21], and our observations in previous gene therapy studies that improved motor function in the rotarod assay was observed only when both the CNS and systemic vector delivery were involved [17], [18], [20]. Further, a recent study also showed improve therapeutic impact in treating MPS IIIB in mice when combining intracranial AAV gene delivery and syngeneic bone marrow transplant, compared to using single approach [21].

Given the fact that the MPS IIIB mouse model strongly resembles the disease in humans, it is reasonable to predict that the neuropathy also manifests the PNS in patients MPS IIIB. It is worth noting that MPS IIIB mice develop characteristic late-stage clinical disorders, such as urine retention, rectal prolapse and/or protruding penis; symptoms which may reflect severe neurological damages [4], [19]. These symptoms are reversible when they begin to emerge at 7–10 mo of age, and then become irreversible within 3–6 weeks, as the disease progresses. Given the profound neuropathology in the DRG, ENS, spinal cord and brain, these late stage neurological disorders may be attributed to the complex neuropathology, including the consequent impairment of both efferent and afferent sensory signal transduction. This may be further supported by the unexplainable frequently recurrent and sometimes severe diarrhea in patients with MPS III [1], [25]. While further studies are needed to investigate the details in PNS neuropathology in MPS IIIB, we anticipate that achieving optimal neurological benefits from therapies will require delivery to both the CNS and PNS in this disease.

Our data have demonstrated neuropathology in the spinal cord of MPS IIIB mice that is global in distribution with differential cell-type presentation, similar to that observed in previous studies in mice and dogs [4], [5]. While the lysosomal storage lesions are observed in a broad range of cell-types throughout the spinal cord, including neurons, microglia, oligodendrocytes, pericytes and ependymal cells, the neuronal targets are predominantly motor neurons in the ventral horn and autonomic neurons in the lateral horn of grey matter. Lysosomal storage pathology does not seem to affect dorsal horn sensory neurons, even though adjacent microglia exhibit profound lysosomal storage lesions. This neuronal-cell-type-specific manifestation of lysosomal storage pathology suggests the function-specific neuronal impacts of heparan sulfates in the spinal cord, especially in efferent signal transduction. This notion is supported by a previous report on differential distribution of heparan sulfate glycoforms in the CNS in MPS IIIB mice, though the mechanisms remain to be investigated. [14] Our data also demonstrated the astrocytosis and microglia activation in the spinal cord, supporting the notion of inflammation in the spinal cord, correlating with immune activation in the brain [6], [11], [12], [26]. Furthermore, it is not surprising to see widespread demyelination in the spinal cord of MPS IIIB mice, given the evidence of oligodendrocyte manifestation, neuroinflammation and neurodegeneration in the brain. While further studies are needed to reveal the detailed mechanisms of spinal cord neuropathy in MPS IIIB, it is highly likely that the aberrant PNS neuropathies, especially damages in DRG, affect afferent sensory signal transduction and may contribute to the functional sensory impairment. Our observation of ENS neuropathology, including lysosomal storage pathology and glial activation in the ENS, strongly supports the hypothesis that the PNS neuropathology is widespread, and that the neurological disease, while predominantly CNS, is likely to be a consequence due to both the CNS and PNS neuropathology.

## Supporting Information

Figure S1 **Distruction of myelin sheath in spinal nerve in MPS IIIB mice.** TEM images of spinal nerve from 1 wt and 2 MPS IIIB mice (6 mo old). Scale bar = 2 µm.
(TIF)
