# Supplementary figures and images for: Peripheral Nervous System Neuropathology and Progressive Sensory Impairments in a Mouse Model of Mucopolysaccharidosis IIIB
Source: PLoS One. 2012 Sep 25;7(9):e45992. doi: 10.1371/journal.pone.0045992 (PMC3457935; doi:10.1371/journal.pone.0045992)

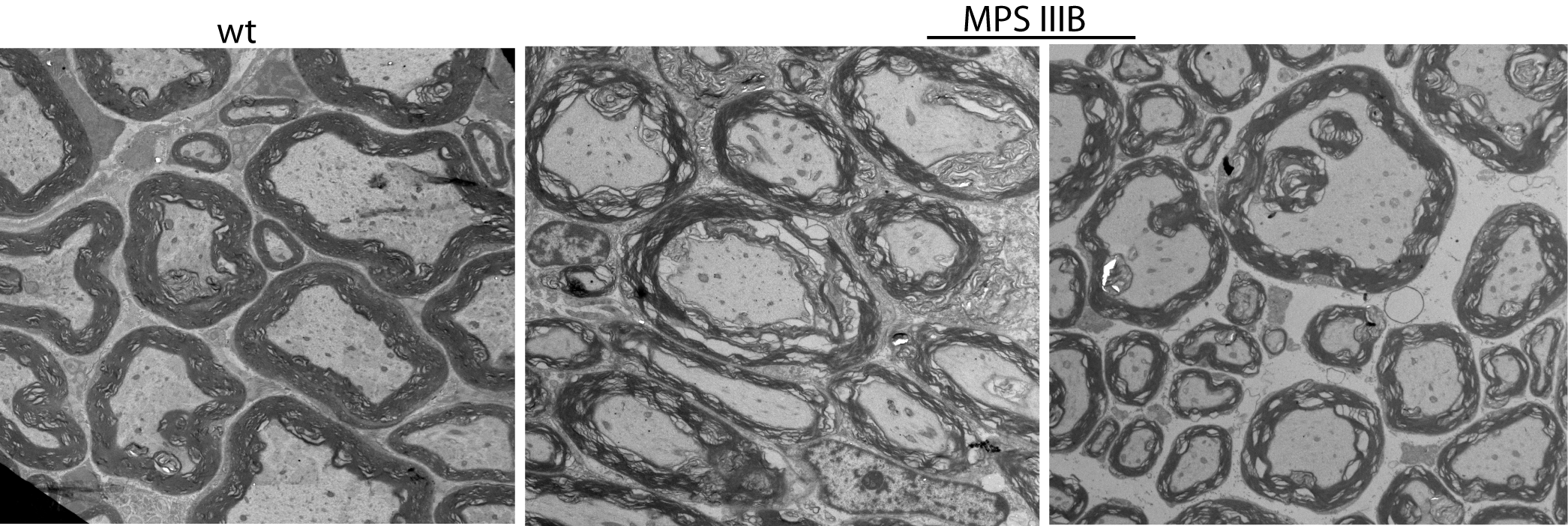

Supplement: Figure S1 — Distruction of myelin sheath in spinal nerve in MPS IIIB mice. TEM images of spinal nerve from 1 wt and 2 MPS IIIB mice (6 mo old). Scale bar = 2 µm. (TIF) [file pone.0045992.s001.tif]
